# Removal Efficiency and Mechanism of Sulfamethoxazole in Aqueous Solution by Bioflocculant MFX

**DOI:** 10.1155/2013/568614

**Published:** 2013-02-17

**Authors:** Jie Xing, Ji-Xian Yang, Ang Li, Fang Ma, Ke-Xin Liu, Dan Wu, Wei Wei

**Affiliations:** State Key Lab of Urban Water Resource and Environment, School of Municipal and Environmental Engineering, Harbin Institute of Technology, Harbin 150090, China

## Abstract

Although the treatment technology of sulfamethoxazole has been investigated widely, there are various issues such as the high cost, inefficiency, and secondary pollution which restricted its application. Bioflocculant, as a novel method, is proposed to improve the removal efficiency of PPCPs, which has an advantage over other methods. Bioflocculant MFX, composed by high polymer polysaccharide and protein, is the metabolism product generated and secreted by *Klebsiella* sp. In this paper, MFX is added to 1 mg/L sulfanilamide aqueous solution substrate, and the removal ratio is evaluated. According to literatures review, for MFX absorption of sulfanilamide, flocculant dosage, coagulant-aid dosage, pH, reaction time, and temperature are considered as influence parameters. The result shows that the optimum condition is 5 mg/L bioflocculant MFX, 0.5 mg/L coagulant aid, initial pH 5, and 1 h reaction time, and the removal efficiency could reach 67.82%. In this condition, MFX could remove 53.27% sulfamethoxazole in domestic wastewater, and the process obeys Freundlich equation. *R* ^2^ value equals 0.9641. It is inferred that hydrophobic partitioning is an important factor in determining the adsorption capacity of MFX for sulfamethoxazole solutes in water; meanwhile, some chemical reaction probably occurs.

## 1. Introduction

In recent decades, the use of pharmaceutical and personal care products (PPCPs) has increased dramatically [1, 2]. They are members of a group of chemicals newly classified as organic microcontaminants in water after pesticide and endocrine disrupting compounds, which stably exist in nature, have properties of being hard-biodegraded, bioaccumulation, and long-range hazardous, posing far-reaching and unrecoverable hazard on ecosystem [3–5]. The presence of PPCPs of emerging concern as increasing evidence suggests their harmfulness [6]. Antibiotic medicine sulfamethoxazole features classic PPCPs, with very low removal ratio in water treatment and high frequency to be detected. In recent decades, although the consume of sulfamethoxazole has been reduced, it is the most popular germifuga in animal food production [7]. It is reported that SMX applied in veterinary directly discharges into the aquatic environment, which has high toxicity [8]. Therefore, there have been large amount of studies on sulfamethoxazole. However, most attention has been focused on identification, fate, and distribution of PPCPs in municipal wastewater treatment plants [9, 10]. It is significant to develop treatment method to remove SMX. The commonly used treatment methods include advanced oxidation process, adsorption, and membrane technology [11–13]. Bioflocculation absorption method has several advantages over other methods, such as going green, being environmentally protective, no second pollution, and being biodegradable [14]. What is more, bioflocculation has been proved to be highly effective and wildly applied, and yet there is no published research on bioflocculation removal of PPCPs. Thus, it is meaningful to study the removal of PPCPs by bioflocculation. Bioflocculant MFX is a metabolized production with good flocculant activity, generated and secreted by *Klebsiella* sp. into the extracellular environment, composed of macromolecular polysaccharide and protein. In this study, based on its physical and chemical property, the effective ingredient of MFX is extracted by water abstraction and alcohol precipitation, transformed into dry powder. And then the removal efficiency and mechanism of sulfamethoxazole in aqueous environment are researched. The study aims at developing an effective treatment method of sulfamethoxazole and expanding the applied range of bioflocculation.

## 2. Materials and Methods

### 2.1. Strains and Media

#### 2.1.1. Bioflocculant-Producing Bacterium, Strain J1:* Klebsiella* sp.   

The strain was screened by our laboratory from activated sludge in municipal wastewater treatment plants and preserved in China General Microbiological Culture Collection Center (CGMCC number 6243).

#### 2.1.2. Inclined Plane Medium (g/L)

Peptone 10, NaCl 5, beef extract 3, agar 15~18, water 1000 mL, pH 7.0~7.2; Flocuclant fermentation medium (g/L): glucose 10, yeast extract 0.5, urea 0.5, MgSO_4_·7H_2_O 0.2, NaCl 0.1, K_2_HPO_4_ 5, KH_2_PO_4_ 2, H_2_O 1000 mL, pH 7.2~7.5.

### 2.2. Methods

#### 2.2.1. Assay Methord

Flocculating rate: 5.0 g chemically pure kaolin clay, 1000 mL tap water, and 1.5 mL 10% CaCl_2_ liquid are added into a beaker, pH is adjusted to 7.2 by adding NaOH, then 10 mL flocculant is added, compared with control without flocculant addition. Flocculator is applied during the experiment, after 40 s fast mixing, and changed into slow mixing for 4 minutes, after 20 min settling, and the absorbance of the supernatant is measured under 550 nm by 721 UV spectrometer [15]. The flocculation efficiency is calculated as follows:

(1) $$\begin{array}{c} \text{flocculation}\;\;\text{efficiency}=\frac{\left(A-B\right)}{A}\times 100\%, \end{array}$$

where *A* is turbidity of the supernatant in control (light transmittance); *B* is turbidity of the supernatant in sample. 

The removal efficiency of sulfamethoxazole is calculated by the following equation:

(2) $$\begin{array}{c} \text{remove}\;\;\text{efficiency}=\frac{\left(C-D\right)}{C}\times 100\%, \end{array}$$

where *C* is the concentration in control and *D* is the sulfamethoxazole concentration after treatment:

 Polysaccharide measurement: Phenol-sulphuric acid method [16].
 Protein measurement: Coomassie light blue [16].

#### 2.2.2. Bioflocculant Preparation

Add 2x volume absolute alcohol (precooled under 4°C) to fermentation liquid, and filter and collect the white flocs after mixing. Add 1x volume absolute alcohol to filtered liquid, and then collect the white flocs again. Add small amount of DI water to collected flocs, after uniformly dissolving, freeze the flocculants in the ultralow temperature freezer for 24 h, and then put them into freeze drying to change the flocculants into dry powder.

#### 2.2.3. Chromatographic Condition

Chromatographic column: C18 (250∗4.6 mm, 5 um); mobile phase is formic acid water: Acetonitrlle (60 : 40 V/V); flow rate 1.0 mL/min; sample size 10 *μ*L; column temperature 30°C; wave length 265 nm [17].

#### 2.2.4. Impact Factor Experiment of Sulfamethoxazole Removal Efficiency

Add flocculants into 1 mg/L sulfamethoxazothe liquid with dosage 0 mL, 1 mL, 3 mL, 5 mL, 7 mL, and 9 mL; set the coagulant aids dosage as 0 mL, 0.5 mL, 1 mL, 1.5 mL, and 2 mL; adjust pH value to 4, 5, 6, 7, and 8 under 5°C, 15°C, 25°C, 35°C, 45°C, and 55°C; change the reaction time as 0 h, 0.25 h, 0.5 h, 0.75 h, 1 h, 2 h, 4 h, 6 h, 8 h, 10 h, and 12 h; calculate the removal rate.

#### 2.2.5. Orthogonal Test of Sulfamethoxazole Removal by Bioflocculants

Based on the preliminary obtained optimum condition, flocculant dosage, coagulant-aid dosage, pH, reaction time, and temperature are considered as influencing parameters. The design of experiment is shown in Table 1.

#### 2.2.6. Adsorption Isotherm Experiment of Sulfamethoxazole Removal by Bioflocculants

Mix the flocculant MFX and sulfamethoxazole with initial concentration as 0.8 mg/L, 1 mg/L, 1.2 mg/L, 1.4 mg/L, 1.6 mg/L, and 1.8 mg/L separately, under different temperature condition as 15°C, 35°C, put on 140 rpm shaking table with constant temperature, conduct adsorption isotherm experiment, and adsorption time is 1 h.

## 3. Results and Discussion

### 3.1. Analysis of Flocculent Active Ingredients

**Figure 1:**
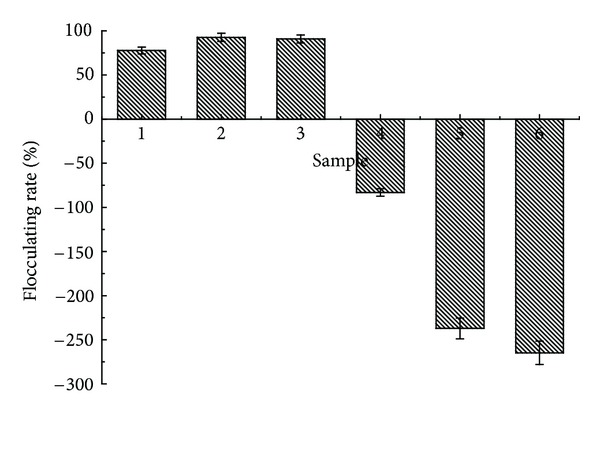
Distribution of flocculent active ingredients.

The strain J1 was short rod-shaped, cream-colored, viscous, smooth, and Gram-positive. J1 was identified as *Klebsiella*. sp on the basis of the morphological characteristics and 16S rDNA sequence. The J1 showed a high yield of flocculant and good flocculation activity toward kaolin suspension. The active ingredients of bioflocculant MFX produced by J1 distributed mainly in the supernatant after the first centrifugation of fermentation broth, that is to say, the flocculation active extracellular secretions remain freely in fermentation broth. (Figure 1). Flocculation ratio after first centrifugation appeared negatively, which proved that the J1 itself was not responsible for the flocculation. The addition of cell suspension leads to the increasing turbidity of raw water. After ultrasonic crushing and centrifugation, bacteria cells were broken and the intercellular content went into the supernatant; the negativity of flocculation ratio showed the fact that the intercellular content may not have flocculation effect. What is more, fermentation broth without inoculation has relatively high flocculation ratio, and it may be caused by the flocculation effect and coagulation aid effect of the phosphate or other inorganic salts. Thus, we may reach the conclusion that the flocculant active ingredient is the metabolized production; in the meantime, the growth medium also contributes to the flocculation. By the isolation and purification of flocculation active ingredients, removing disturbance of growth medium, a further conclusion may be reached; that, is the active ingredient is the secondary metabolites of bacteria fermentation (extracellular polymeric substances (EPS)).

Table 2 presents MFX that has apparent results in the saccharides and protein chromogenic reactions. According to Table 2, we can conclude qualitatively that the major ingredients of the flocculant produced by J1 are polysaccharides and protein, the polysaccharides content is 0.0656 mg/mL, and the protein content is 0.1021 mg/mL.

After the enzymatic digestion of EPS, polysaccharides were removed, while proteins remained. The proteins accounted for 15.05% (cellulase), 61.9% (*α*-amylase), and 11.4% (*β*-amylase) of the flocculation activity. On the contrary, proteins were removed, while polysaccharides remained. EPS has no flocculent activity (Table 3). Obvious decrease in flocculation activity was observed after the flocculant MFX was exposed at 70°C for 20 min, indicating that it was low thermostable. This implies that the active constituents in MFX were proteins and polysaccharides, and proteins dominant accounted for the flocculation activity.

### 3.2. The Impact Factors on Removal Efficiency of Sulfamethoxazole by MFX

**Figure 2:**
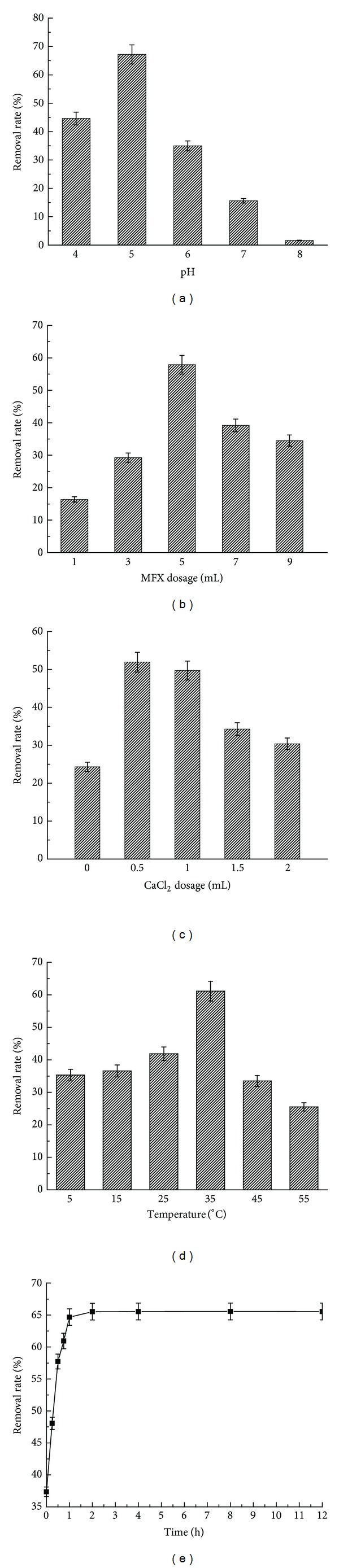
The influence of ecological factor on removal rate. (a) is the influence of pH; (b) is the influence of MFX dosage; (c) is the influence of CaCl_2_ dosage; (d) is the influence of temperature; (e) is the influence of time on removal rate.

Five parameters: pH value, flocculant dosage, coagulation aid ratio, flocculation time, and temperature are measured to see the effects of these factors on sulfamethoxazole removal efficiency. Along with the changes of pH value, the removal efficiency increases firstly then decrease, and changes sharply, from where we could know that pH does affect removal ratio a lot. It is shown in Figure 2(a) that, between pH 4 and 5, the removal efficiency increases along with pH increment. When pH is 5, MFX possesses the strongest flocculation capacity and the highest flocculation efficiency, which is 67.2%. Between pH 6 and 8, the removal efficiency falls steeply when pH is 8, and the removal efficiency is only 1.61%. The result demonstrated that the bioflocculant has higher removal efficiency on sulfamethoxazole in the acidic condition, while the alkali condition results in relatively poor removal efficiency.  Figure 2(b) shows that along, with the increase of flocculant dosage, the removal efficiency increases firstly, then decreases, and changes acutely. When flocculant dosage varies within the range from 1 mL to 5 mL, the removal efficiency improved with the increasing dosage. When flocculant dosage is 5 mL, the optimum removal efficiency is obtained, which is 57.89%. As seen in Figure 2(c), the dosage of coagulant aid also has impact on the removal efficiency. Increasing volume ratio of coagulant aid leads to increasing removal efficiency. When coagulant aid dosage is 0.1 times of flocculation dosage, which is 0.5 mL, more than 50% removal efficiency is reached. Afterwards, the increment of coagulant aid dosage decreases flocculation capability and removal efficiency. In the meantime, the removal efficiency is around 20% without coagulant aid addition, which proves that bioflocculant could remove sulfamethoxazole without coagulant aid.  Figure 2(d) shows that the removal efficiency increases firstly, then decreases with the change of temperature, but within narrow fluctuation range. When temperature is between 5°C and 25°C, the removal efficiency increases slowly. When temperature is 35°C, the strongest flocculation capability is obtained and the highest removal efficiency is reached, which is 67.20%. The removal efficiency decreases on the temperature of 45°C and 55°C. According to Figure 2(e), the removal efficiency increases exponentially within the first 30 minutes, after 30 minutes the increment slows down and remains the same after 1 hour, reaching equilibrium. This is because of that a large amount of adsorbate exists in the liquid in the beginning of the reaction and is adsorbed quickly by adsorbent. With the occupation of the adsorption sites, adsorption quantity inclines slowly. After equilibrium is reached, the adsorption quantity remains constantly.

In conclusion, the optimum flocculation condition is pH 5, flocculant dosage 5 mL, coagulant aid dosage 0.5 mL, flocculant reaction time 1 h, and temperature 35°C. Under this condition, the highest removal efficiency is reached, which is 67.82%. It is reported that the removal efficiency using conventional water treatment process including preoxidation, coagulation, and sand filtration is 36% [18], and the removal efficiency by microelectrolysis Fentonis is 65.5% [19]. It can be seen that bioflocculant is obviously more efficient than normal treatment.

### 3.3. The Removal Efficiency of Sulfamethoxazole in Domestic Wastewater by MFX

In order to evaluate the removal effect of bioflocculant MFX on sulfamethoxazole in actual wastewater, domestic wastewater is used, with sulfamethoxazole concentration as 23.26 ug/L. 9 sets of experiments are conducted according to the orthogonal design table L9 (3^4^), and results are shown in Table 4.

As is shown in Table 4, according to average value analysis, optimum flocculation condition is the combination of A1B3C2D2, which is pH 5, flocculant dosage 8 mL, coagulant aid dosage 0.2 mL, and reaction time 1 h. It has been proved that, under this condition, the removal efficiency of sulfamethoxazole is 53.27%.

By comparing the extremums, we may reach a conclusion that the effect degrees of factors obey the following order: RA > RB > RC > RD. That is to say, pH value affects mostly, followed by flocculant dosage, coagulant aid dosage, and reaction time. This is because that the dissociation of flocculant occurs within a certain pH range. Proper pH value could increase the dissociation degree, lead to a higher charge density of flocculant, benefits the spreading of the flocculant molecules, and facilitates the bridging action of the bioflocculant. Thus, pH value plays a critical role [20]. When the flocculant dosage is relatively low, early adsorption saturation may be reached, the removal efficiency of contaminants decreases. When flocculant dosage is high, extraflocculant weakens the bridging effect due to adsorption sites overlapping and finally affects the removal efficiency of specific contaminant. Thus, proper flocculant dosage plays an important role in affecting removal efficiency. Some studies show that metal ions addition could change the surface charge of colloids. sulfamethoxazole flocs in the water are negatively charged, and when approaching positively charged flocculant hydrolyzates and calcium ions in coagulant aid, charge neutralization occurs on the surface of sulfamethoxazole and makes the colloidal particles to sediment, exacerbating the collision of colloids and collision between colloids and flocculant, integrating a whole group under Van der Waals' force, finally precipitating from water by gravity [21].

In the research of removal mechanism of sulfamethoxazole aqueous solution, the highest removal efficiency is more than 60%, but, in the removal of sulfamethoxazole in wastewater, the highest removal efficiency under optimum condition is only 53.27%. This may be caused by the relatively low concentration of sulfamethoxazole in wastewater, which is 23.26 ug/L. The limitation of measurement methods may lead to potential systematic errors. On the other hand, domestic wastewater has complex component, and there exists reversible and irreversible competitions among substrates, liming the combination of flocculant and sulfamethoxazole. What is more, some unknown ions and organic compounds may also decrease the removal efficiency.

### 3.4. The Mechanism of Sulfamethoxazole in Aqueous Solution by MFX

**Figure 3:**
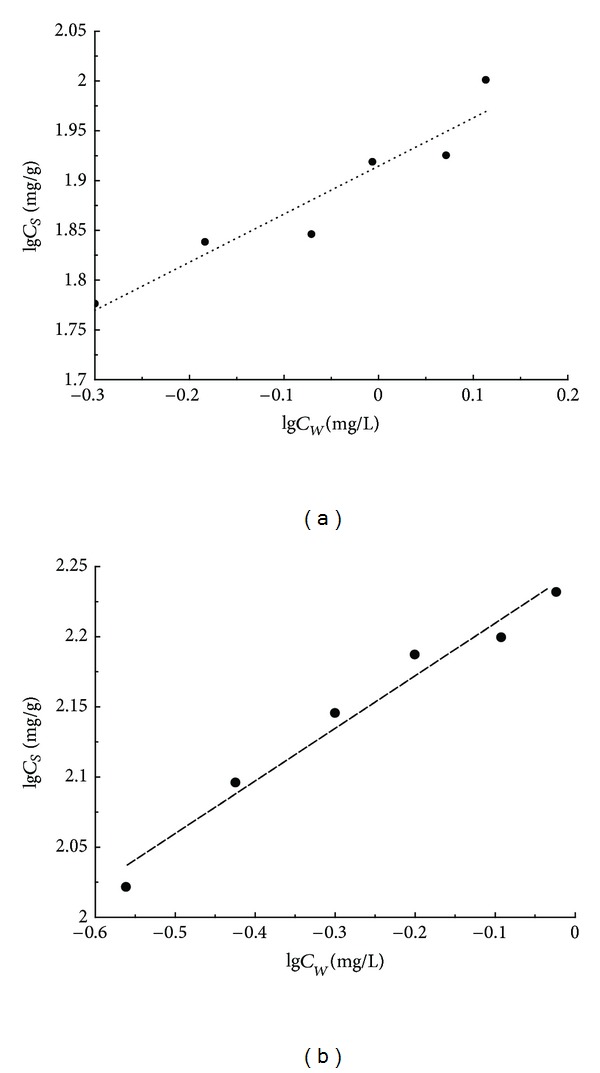
Adsorption isotherms of sulfamethoxazole by MFX adsorbent in 15°C (a) and 35°C (b).

The dry power of MFX is white, sparses and reticulate, while the aqueous solution is milky white, ropy, and turbid. The material of MFX adsorbent is glycoprotein which has hydrophobicity and displays a wide range of sorption behavior for hydrophobic organic compounds in aqueous solutions [22]. The adsorption isotherms of sulfamethoxazole on MFX adsorbents are presented in Figure 3 and Table 5. Adsorption data are fitted to Freundlich isotherm model which is the most widely used models for describing adsorption phenomena in aqueous solutions. The Freundlich isotherm model is an empirical equation accurate for describing adsorption in aqueous solutions at low solute concentrations. Expressions and interpretations are as follows. The maximum adsorption capacity of the adsorbent is represented by *K*_*F*_ (mg/g), while *n* is constant which is indicative of the adsorption energy and intensity:

(3) $$\begin{array}{c} C_{S}=K_{F}\cdot C_{W}^{1/n}\\ \text{lg}C_{S}=\frac{1}{n}\text{lg}C_{W}+\text{lg}K_{F}, \end{array}$$

where *C*_*S*_ is mass concentration in solid phase after adsorption equilibrium (mg/g); *C*_*W*_ is concentration in liquid phase after adsorption equilibrium (mg/L).

The results show that MFX exhibited high-adsorption capacities for sulfamethoxazole in water. A maximum adsorption capacity (*K*_*f*_) of 176.6445 mg/g was obtained with MFX in 35°C. Compared Figure 3(a) with Figure 3(b), it can be perceived that linear correlation is marked in 35°C. According to *n* value, it is known that, under this temperature, the adsorptive property of MFX to sulfamethoxazole is high. However, MFX has poorer adsorption efficiency in 15°C. *R* ^2^ value is only 0.882, while n value is lower than the former.

This is due to the effect of temperature on chemical reaction and molecular movement, which finally promote or restrain flocculent effect. On the one hand, providing appropriate temperature, colloidal particle is bombarded intensively by molecules of flocculation to form a whole. If temperature is excessively low, molecular movement slows down, and reaction rate lessens, leading to bad adsorptive property. On the other hand, some active groups between bioflocculant and sulfamethoxazole start the chemical reaction to separate out of aqueous solution system. What is more, when hydrophobic chain polymer-bioflocculation MFX comes into sulfamethoxazole aqueous solution system, with the help of appropriate pH and Ca^2+^, sulfamethoxazole becomes unstable rapidly, passing into flocculation phase.

Driven by such mechanism, the adsorption on MFX does not rely on a high porosity and a resultant high specific surface area to reach a high adsorption capacity, as for most activated carbon and polymeric adsorbents. This result implies that hydrophobic partitioning is an important factor in determining the adsorption capacity of MFX for sulfamethoxazole solutes in water; meanwhile, some chemical reaction probably occurs.

## 4. Conclusion

This work demonstrates the efficient removal of sulfamethoxazole from water using bioflocculant MFX as adsorbents. The findings are summarized as follows.

1. The active flocculent constituents of MFX are EPS which is composed by polysaccharides and proteins fermented by J1. Proteins mainly accounted for the flocculation activity.
2. The MFX displays great sorption behavior for sulfamethoxazole in aqueous solutions. The optimum condition is 5 mg/L bioflocculant, 0.5 mg/L coagulant aid, initial pH 5, and 1 h reaction time, and the removal efficiency could reach 67.82%.
3. Using MFX, the removal rate of sulfamethoxazole in domestic wastewater can reach 53.27%. The effect size of ecological factor is as follow: pH > flocculant dosage > coagulant aid > reaction time.
4. It is efficient for MFX to remove sulfamethoxazole in aqueous environment in 35°C. And the process obeys Freundlich equation. *R* ^2^ value equals 0.9641. It is inferred that hydrophobic partitioning is an important factor in determining the adsorption capacity of MFX for sulfamethoxazole solutes in water.

The study shows that bioflocculation MFX can be used as an efficient alternative adsorbent for the removal of sulfamethoxazole in water, with high-adsorption capacities observed in actual wastewater. Further studies are underway to make mathematical models of the relationship between flocculant and contaminant. When many PPCPs coexist, the research on removal efficiency with bioflocculant is more significant.

## Figures and Tables

**Table 1:** Factors and levels of orthogonal test.

Levels	*A* pH	*B* Flocculant dosage (mL)	*C* Coagulant aid dosage (mL)	*D* Reaction time (h)
1	5	2	0	0.5
2	6	5	0.2	1
3	7	8	0.5	1.5

**Table 2:** Qualitative analysis of flocculent active ingredients.

Reaction type	Analytical method	Phenomenon
Polysaccharide	Molish reaction	+
	Anthrone reaction	+
	Seliwanoff reaction	−

Protein	Ninhydrin reaction	+
	Biuret reaction	+
	Xanthoprotein reaction	+

**Table 3:** Enzymatic digestion of flocculent active ingredients.

Reaction type	Enzym	Flocculation rate after enzymatic digestion	Floc
Polysaccharide	Cellulase	15.05%	Small
	*α*-amylase	61.90%	Big
	*β*-amylase	11.4%	Small

Protein	Trifluoroacetic acid	Negative	None
	Pepsase	Negative	None
	Trypsin	Negative	None

**Table 4:** Orthogonal test result and visual analysis.

Tests	*A* pH	*B* Flocculant dosage (mL)	*C* Coagulant aid dosage (mL)	*D* Reaction time (h)	Removal efficiency (%)
1	1	1	1	1	40.64
2	1	2	2	2	48.38
3	1	3	3	3	50.13
4	2	1	2	2	36.91
5	2	2	3	1	30.26
6	2	3	1	3	44.27
7	3	1	3	2	29.86
8	3	2	1	3	35.03
9	3	3	2	1	41.70
Average 1	46.383	35.803	39.980	37.533	
Average 2	37.147	37.890	42.330	40.837	
Average 3	35.530	45.367	36.750	40.690	
Variance	10.853	9.564	5.580	3.304	

**Table 5:** Freundlich isotherm parameters at different temperature.

*T* (°C)	Freundlich equation	*K*_*F*_	*R* ^2^	*n*
15	*y* = 0.483*x* + 1.9146	82.1486	0.8820	2.07
35	*y* = 0.3748*x* + 2.2471	176.6445	0.9641	3.18
